# A 75-Year-Old Woman with a Hemispheric Stroke

**DOI:** 10.1371/journal.pmed.0020079

**Published:** 2005-04-26

**Authors:** Stavros K Kakkos, George Geroulakos

## Abstract

What are the causes, investigation, and management of hemispheric stroke? Find out in this case-based article

## DESCRIPTION of CASE

A 75-year-old right-handed woman presented with a two-day history of symptoms suggestive of a right hemispheric stroke (slurred speech and left facial and left arm weakness). She had no previous cerebrovascular symptoms, such as symptoms of a previous transient ischaemic attack or amaurosis fugax (loss of vision in one eye due to a temporary lack of blood flow to the retina). Past medical history included long-standing hypertension and chronic obstructive pulmonary disease. She was on amlodipine, 10 mg once daily, and salbutamol and fluticasone inhalers.

On examination, the patient had a Glasgow Coma Score of 15, she was apyrexial, her pulse rate was regular, at 80 per min, and her blood pressure was 176/99 mm Hg. There was no cardiac murmur or carotid bruits. She had left-sided weakness.

### What Investigation Is Indicated at This Stage?

Brain imaging is necessary for two main reasons. The first is to exclude a brain haemorrhage (responsible for 25% of all strokes [1]); against this diagnosis was the absence of headache and a normal Glasgow Coma Score. The second is to rule out a brain tumour.

**Figure 1:**
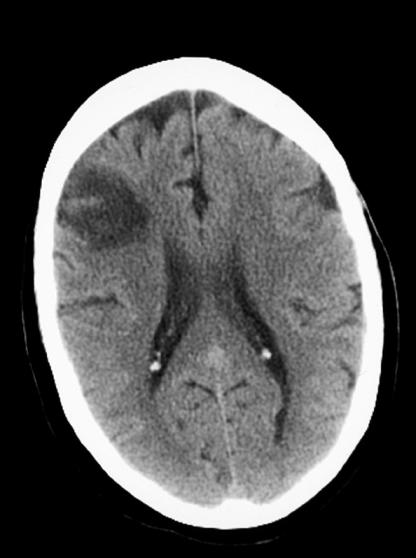
CT Brain Scan on Admission Showing an Infarct in the Posterior Right Frontal Lobe

Computed tomography (CT) brain scanning on admission showed two areas of low density within the right cerebral hemisphere, one in the right parietal lobe and one in the posterior right frontal lobe (Figure 1), most likely ischaemic in nature. Small low-density lesions consistent with lacunar infarcts were also seen in both basal ganglia, the most prominent ones seen within the left basal ganglia. There was also marked frontal atrophy, and atrophy of the brain stem structures. The CT scan showed no evidence of haemorrhagic transformation of the infarct, a condition that is a contraindication for anticoagulation.

Routine blood tests (full blood count, urea and electrolytes, and clotting), an electrocardiogram, and a chest X ray were performed before the CT brain scan. The electrocardiogram showed no arrhythmia or changes suggestive of an old or new myocardial infarction. The normal electrocardiogram raised the possibility of embolisation from a large artery (such as the right carotid artery or the aortic arch), rather than from the heart.

### How Did We Identify the Source of Embolisation?

**Figure 2:**
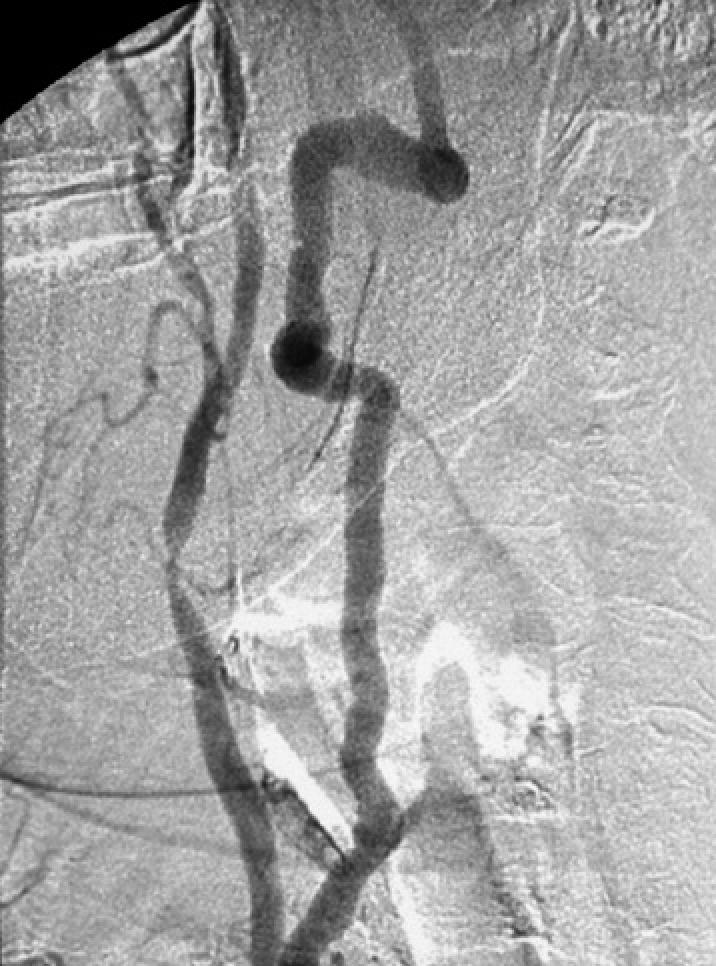
Digital Subtraction Angiography Showing a Tight Stenosis of the Distal Right Common Carotid Artery

A carotid ultrasound showed a calcified, haemodynamically significant plaque at the right carotid bifurcation. A similar lesion seen at the left carotid bifurcation was not haemodynamically significant. Carotid angiogram showed a tight stenosis of the distal right common carotid artery (Figure 2), and occlusion of the left internal carotid artery.

### What Was the Management Plan at This Stage?

Bendrofluazide, 2.5 mg once daily, and perindropril, 2 mg once daily, were prescribed to treat the patient's hypertension. Antiplatelets (aspirin, 75 mg once daily, and clopidogrel, 75 mg once daily) and a statin (simvastatin, 40 mg at night) were also prescribed. Carotid endarterectomy was scheduled. Bisoprolol, 2.5 mg once daily, was prescribed to reduce the risk of periprocedural myocardial ischaemia [2].

The patient's symptoms gradually improved and she was discharged two days later. A contrast-enhanced CT brain scan performed nine days after the onset of symptoms confirmed the previous findings. The dose of perindopril was gradually increased from 2 mg to 6 mg daily to achieve satisfactory control of the patient's hypertension.

### After a Hemispheric Stroke, How Soon Should Carotid Endarterectomy Be Performed?

Six weeks after the stroke, the patient underwent elective right carotid endarterectomy under general anaesthesia with the use of a shunt. Although some surgeons favour early carotid endarterectomy, most agree that this should not be performed earlier than six weeks, to allow the autoregulative mechanism of the brain to recover [3,4]. Arteriotomy was closed with a Dacron patch. No neurological deficits or cranial nerve palsy were noted postoperatively. The patient's discharge was postponed until the seventh postoperative day because of a mild urinary tract infection and an episode of syncope. Histology revealed an atheroma producing near total lumen occlusion. Eleven months after the operation no new neurological events have occurred.

## DISCUSSION

### Causes of Hemispheric Ischaemic Stroke

Mohr et al. classified the causes of ischaemic stroke into three broad categories: embolism to the brain of cardiac or aortic origin, cerebral ischaemia due to perfusion failure and artery-to-artery embolism, and cerebral artery thrombosis [5]. Embolism to the brain of cardiac or aortic origin includes myocardial infarction, atrial fibrillation, valvular disease in native, prosthetic, or repaired cardiac valves (including mitral valve prolapse), embolism of aortic arch origin, and myxoma of the heart and from venous thrombi via a patent foramen ovale. Cerebral ischaemia due to perfusion failure and artery-to-artery embolism includes large artery atherosclerotic plaque, vasculitis, and other arterial disease and small artery occlusion. Thrombosis is caused by prothrombotic states.

Carotid stenosis accounts for about 20% of all cases of ischaemic stroke [6], and is considered as the single most preventable cause of stroke. Like all atherosclerotic diseases, the most common risk factors for carotid stenosis are smoking, hypertension, hyperlipidaemia, and diabetes mellitus.

### Investigation of Carotid Artery Bifurcation Stenosis

Carotid ultrasound duplex is the imaging method of choice for the initial investigation of a patient with suspected carotid artery stenosis [7]. It is non-invasive and low-cost, and can be easily repeated if necessary.

**Figure 3:**
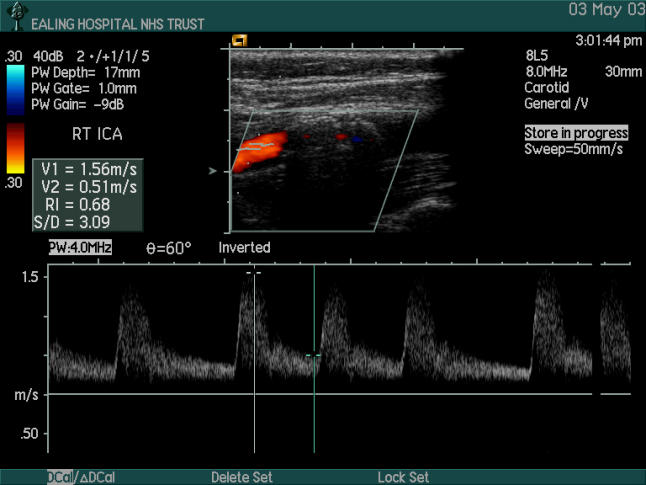
Carotid Artery Ultrasound Showing a Completely Calcified Atherosclerotic Plaque (Geroulakos Type 5) Although post-stenotic velocities are increased, accurate grading with ultrasound is not possible.

Anatomical criteria at the point of stenosis (cross-sectional area reduction or diameter reduction) should always be applied to ensure that stenosis is present and that flow velocity is not increased secondary to a vessel curve. A limitation of using anatomical criteria to estimate the degree of carotid stenosis is that in the presence of heavy acoustic shadowing (due to calcification) interrupting flow visualisation, no intraluminal diameter reduction can be calculated (Figure 3).

**Figure 4:**
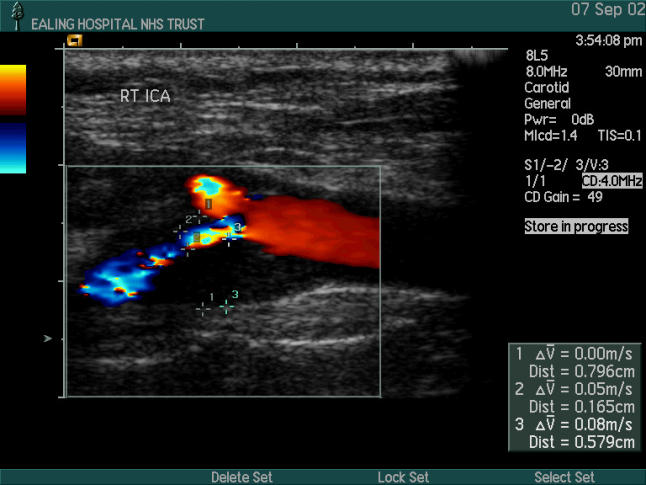
Carotid Artery Ultrasound Showing an Echolucent Atherosclerotic Plaque (Geroulakos Type 2) This plaque is causing approximately 80% stenosis, as determined using diameter reduction and velocity ratio methods.

Because of these problems, intrastenotic velocity measurements are widely used; in cases of acoustic shadowing due to calcification, these measurements should be performed just distally to the acoustic shadowing, at the point of the flow jet. Long acoustic shadowing, known also as the Gibraltar sign [8], can result in falsely reduced velocity measurements and downgrade the stenosis. It has been found that velocity measurements are affected by different ultrasound scanners, physiological changes, and the presence of contralateral occlusion or “tandem” lesions [9]. To overcome this problem, intrastenotic flow velocities are “normalised” using the common carotid or distal internal carotid flow velocities as a reference point, and the resulting ratio is used. There are potential problems related to the sampling point and to patients with heart failure with falsely low end-diastolic velocity of the common carotid artery [10]. In order to overcome potential limitations of individual criteria, the use of combined criteria has been suggested (Figure 4) [11,12].

**Figure 5:**
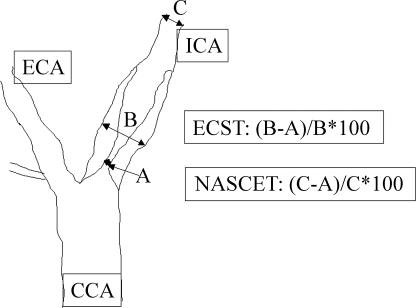
Angiographic Methodology of Grading Carotid Stenosis Grading can be done in relation to the carotid bulb (ECST method) or the distal internal carotid stenosis (NASCET method). CCA, common carotid artery; ECA, external carotid artery; ICA, internal carotid artery.

Selective arteriogram is nowadays rarely indicated, because the procedure itself can cause stroke, and non-invasive alternative methods (magnetic resonance angiography) are available. There is no consensus on the optimum method to grade carotid stenosis (Figure 5) [13,14].

## The Role of Carotid Endarterectomy in Preventing a Recurrent Event

Carotid reconstruction was first performed by Eastcott et al. at St. Mary's Hospital, London, in 1954 [15]. However, it took nearly four decades until trial evidence became available to show that carotid endarterectomy was better than best medical treatment in patients with amaurosis fugax or hemispheric symptoms, transient ischaemic attacks, or stroke who had made a good recovery and whose symptoms were caused by severe carotid bifurcation stenosis (>70% with the North American Symptomatic Carotid Endarterectomy Trial [NASCET] method or >80% with the European Carotid Surgery Trial [ECST] method) [13,14]. The two-year risk of stroke in the medical arm of NASCET was 26% compared with 9% in those who underwent endarterectomy [13]. Subsequently, the NASCET trialists reported that endarterectomy reduces the five-year risk of stroke in moderate stenosis (50%–69%) from 22.2% to 15.7% [16]. Longer follow-up also showed that the long-term risk of stroke after carotid endarterectomy is about 1% per year. A recent meta-analysis of the NASCET and ECST trials showed that benefit from surgery was greatest in men, patients aged 75 years or older, and those randomised within two weeks after their last ischaemic event, and fell rapidly with increasing delay [17].

Surgery is usually performed at six weeks if there is good recovery, but there is a tendency to perform it earlier in patients with transient ischaemic attacks or strokes with good recovery when CT brain scan shows no infarct. Surgery reduces the risk of stroke by 50% even if the event occurred more than six months previously, as shown by the Medical Research Council Asymptomatic Carotid Surgery Trial [18], but because of the low incidence of stroke at five years and the relatively small number of patients in the trial, benefit was only marginally significant [18].

### Best Medical Treatment

While recovering from stroke and awaiting carotid endarterectomy, aspirin even at a low dose of 75 mg daily reduces the risk of recurrence. This is improved with dipyradamole [19], but not clopidogrel [20]. The Heart Protection Study has recently shown that regardless of pre-treatment lipid levels, lipid-lowering agents are beneficial in secondary prevention of stroke [21]. In our patient's case, total cholesterol levels after treatment were 3.6 mmol/l.

### Carotid Endarterectomy under Local Anaesthesia, or Carotid Angioplasty and Stenting?

Minimally invasive treatment is considered nowadays a preferable mode of delivering health care, and carotid disease is no exception. Meta-analysis of previous studies has shown that carotid endarterectomy under local anaesthesia has less surgical hazards than under general anaesthesia. This was the basis for the GALA trial—a multicentre randomised trial assessing the relative risks of stroke, cardiac events, and death with these two different treatments (http://www.galatrial.com/). The results are not yet available.

**Key Learning Points**

- Although carotid stenosis accounts for about 20% of all cases of ischaemic stroke, it has been considered as the single most preventable cause of stroke.
- Carotid ultrasound is the method of choice for the initial investigation of a patient with suspected carotid artery stenosis.
- Carotid endarterectomy has been proven to reduce the incidence of recurrent stroke, mainly in severe stenosis.
- Carotid stenting has recently emerged as an alternative procedure.
- Early intervention as soon as possible is desirable to reduce the risk of recurrent stroke.

Carotid angioplasty and stenting with the aid of distal protection devices has recently emerged as a good alternative to endarterectomy, being equivalent or slightly better, when periprocedural complications are considered [22,23]. In the SAPPHIRE trial, performed in selected high-risk patients, those allocated to the stenting group had fewer cardiovascular events than those undergoing surgery [23].

## Abbreviations

CT: computed tomography
ECST: European Carotid Surgery Trial
NASCET: North American Symptomatic Carotid Endarterectomy Trial
